# Interpreting Bibliometric Data

**DOI:** 10.3389/frma.2020.628703

**Published:** 2021-02-09

**Authors:** Martin Szomszor, Jonathan Adams, Ryan Fry, Chris Gebert, David A. Pendlebury, Ross W. K. Potter, Gordon Rogers

**Affiliations:** ^1^ Institute for Scientific Information, Clarivate, London, United Kingdom; ^2^ Institute for Scientific Information, Clarivate, Philadelphia, PA, United States

**Keywords:** bibliometrics, responsible metrics, data interpretation, research assessment, research policy

## Abstract

Many academic analyses of good practice in the use of bibliometric data address only technical aspects and fail to account for and appreciate user requirements, expectations, and actual practice. Bibliometric indicators are rarely the only evidence put before any user group. In the present state of knowledge, it is more important to consider how quantitative evaluation can be made simple, transparent, and readily understood than it is to focus unduly on precision, accuracy, or scholarly notions of purity. We discuss how the interpretation of ‘performance’ from a presentation using accurate but summary bibliometrics can change when iterative deconstruction and visualization of the same dataset is applied. From the perspective of a research manager with limited resources, investment decisions can easily go awry at governmental, funding program, and institutional levels. By exploring select real-life data samples we also show how the specific composition of each dataset can influence interpretive outcomes.

## Introduction

In this paper, and in the context of good and responsible research evaluation, we review the challenge of making correct use and interpretation of the rich information on research activities and outcomes that can be mined from the data around academic journal publications and their citations. This challenge exists at three levels. First, summary citation metrics are usually insufficient to enable fully informed interpretation by the intended users, who are typically research experts in their own fields but unfamiliar with the nature of these data. Second, bibliometric analysis is a tool, the good use of which depends on the user and on the context, and it sharpens questions more often than provides answers (Moed, 2020). Third, because alternative visualisations supporting better interpretation require additional work by these users, they often default to simpler metrics because of time pressure.

We consequently suggest that the priority around scientometric research and practice is not about academic development, which has been extensive over the last few decades, but about practical user focus. There is a need for a structured change in describing how best to use bibliometric analysis. The user needs to be able to start from the context of application with an evaluation framework within which they can specify the data and analytical tools relevant to the questions they pose. The user needs to access information that enables an *a priori* understanding of how they will use these tools, so bibliometric researchers must understand and consider the user perspective. And the user needs to see data presented not as summary point metrics but in a form that allows accessible, interpretive exploration. We examine these challenges through analyses of international research activity and performance.

## Origins

It is widely acknowledged that bibliometric indicators have become one of the most frequent tools of normal practice in evaluative research management. The development of research evaluation practices has been well documented as it shifted from structured processes drawing on strong peer review (Gibbons and Georghiou, 1987) through strategic reorientation (Georghiou, 1995) to systems increasingly drawing on data and metrics (Martin, 1996; Adams et al., 2007; Hicks, 2010; Hicks and Melkers, 2012. Bibliometric indicators, which had been of a specialist nature prior to 1990 (e.g., Narin, 1976; Martin and Irvine, 1983), were introduced to a wider audience during the 1990s when the former Science Citation Index became accessible on-line as the *Web of Science*™ (WoS). Data used for national comparisons of research performance were published in widely-read journals (May, 1997; Adams, 1998) that brought them to the attention of a larger audience who applied them to institutional, program and policy purposes.

Research evaluation may be seen as a reflection of a broader societal shift to institutional managerialism and public sector accountability. As Langfeldt et al. (2020) note: “mechanisms for constituting research quality notions that were once reserved for highly professionalized knowledge communities have extended to encompass notions generated within policy and funding domains.” It was spawned by a growth in research and tertiary education systems that was more rapid than the growth of resources that governments were willing to allocate. For the United Kingdom, as an example with which we have particular familiarity, most projects submitted to Research Councils in the 1970s and peer reviewed as ‘alpha - fundable’ could be financially supported. Then the proportion funded began to fall, so new but still peer-selective criteria were introduced (alpha 1–5). At the same time the country faced an economic and energy crisis, so wider resource constraints appeared. The bodies responsible for funding research in United Kingdom universities (general grants via the University Grants Committee, project grants via the Advisory Board for the Research Councils) reported on the need for selectivity in research distribution (UGC, 1982; ABRC, 1983; UGC, 1984). Thus, the first national Research Selectivity Exercise was introduced in 1986 and led to a more formalized and structured Research Assessment Exercise (RAE) from 1992.

Such an exercise as the RAE had a profound effect on the strategic view of the research enterprise, the management of research in universities, and the spotlight thrown on the individual researcher. The United Kingdom’s procedures also attracted widespread international attention, if not always emulation. It also produced a formidable workload for assessment panel members, who had other full-time roles in addition to the peer review work. Analysis of the results of successive RAEs in 1992 and 1996 were soon augmented with the more accessible bibliometric data then available and thus attention inevitably turned to the idea that quantitative analysis might substitute for some of the onerous qualitative review. After RAE2001, the proposal for a ‘metrics based’ review process was brought under serious central review (Roberts, 2003) but rejected after a pilot exercise prior to RAE2008 (Evidence, 2009).

The United Kingdom’s experience of assessment and metrics’ policy was reflected elsewhere in Europe, notably in the Netherlands and Scandinavia and, in due course, the ideas spread (Sivertsen, 2017). As a consequence, research evaluation using, to a very variable degree, some form of publication and/or citation data is now widespread and present in different forms and at various levels in for example: European programs (European Science Foundation, 2012), in Australia (ARC, 2019), Finland (Lahtinen et al., 2005), Italy (CIVR, 2006; Abramo and D’Angelo, 2015), New Zealand (Buckle and Creedy, 2019; PBRF, 2020), Sweden (Karolinska Institute, 2010), Spain (Jiménez-Contreras et al., 2003), Norway (Sivertsen, 2018), the United Kingdom (REF, 2020) and the United States (National Institutes of Health, 2008). Thomas et al. (2020) recently reviewed 350 research papers on performance-based research evaluation arrangements and discuss important limitations in applying and using such research.

### Problems

Jappe et al. (2018) noted that there is a gap between the demand for indicator-based performance assessment by research organizations and the researchers being assessed. Researchers - and their works - come from a multi-modality of disciplines and cultures with their own norms and expectations. However, because the academic sector, at discipline level, has taken little or no responsibility for understanding and interpreting quantitative indicators based on citation data, *de-facto* and generic standards of research excellence have been defined at system level by others (including scientometricians and data providers) without being challenged by the implied authority of the domain experts. While the possible forms of analysis are diverse, Jappe (2020) reviewed 138 evaluation studies from 21 EU countries, covering the period 2005 to 2019, and found that bibliometric research assessment, which was common to the United Kingdom, the Nordic network, the Netherlands and Italy, was most frequently based on ‘citation impact’ metrics, usually with reference to international scientific fields.

The most widely used standard indicator for ‘citation impact’ is the number of citations received by a publication, normalized “with reference to international scientific fields” (Jappe, 2020). It is generally understood that papers with higher citation counts are associated with greater influence or ‘impact’ since they reflect acknowledgment by other researchers (Garfield, 1955). Citation counts have in turn been shown to be correlated with other indicators of research performance, such as peer review (Evidence, 2007; Waltman, 2016; Aksnes et al., 2019).

To this simple summary several essential caveats must be made. First, the citation metric is only an indicator of impact. Citation counts reflect impact indirectly through a general population relationship and, for individual papers, may be awry in their information. Indeed, the mantra ‘on average’ has wide applicability to every aspect of this kind of analysis. Second, citation counts rise over time, older papers have more citations ‘on average’ than more recent and an adjustment must be made to take account of the years since publication. Third, citations accumulate at rates that are field dependent. For example, life sciences are more prolific and exhibit higher rates of citation on average than technological and social sciences and an adjustment must be made to take account of the field of publication (Moed et al., 1985a). Fourth, document type affects citation rates with reviews in journals cited more often than articles (‘on average’, see Ketcham and Crawford, 2007; Miranda and Garcia-Carpintero, 2018) while conference proceedings are cited less often than journal papers.

The ‘standard indicator’ (the observed document citation count) is therefore processed before analysis. It is, usually, compared to the global average (or ‘expected’) count for the same document type, year of publication and field. Field is usually determined from a pre-set categorical structure which, for WoS, is based on journal assignment to discipline-based categories. Then, the ratio of observed/expected citation counts is used to calculate an average Category Normalized Citation Impact (CNCI) for a research group, institution or country. Again, recall that this CNCI value is an indicator, not a metric, and is now at some distance from the target research activity under evaluation.

So, this general procedure refers to a simple index, inferred to be a reasonable indicator of other aspects of research performance for larger samples (Rogers et al., 2020), that may or may not be relevant to the research objectives that are the proper target of an evaluation. For the humanities, citation counts are of little informational value and indeed journal articles are usually secondary to monographs as a signal of intellectual significance. For applied research of industrial or policy significance, value is reflected in utility and application, not in later academic references. Even where citations are a more appropriate currency, the basic caveats recognized long ago (Moed et al., 1985b), along with a large number of more nuanced issues of qualification (Pendlebury, 2009), are not universally understood by the domain-expert users and their research managers. This leads to extensive misuse (Moher et al., 2018) and consequent reaction from researchers and observers (DORA, 2012; Hicks et al., 2015; Wilsdon et al., 2015).

### Users and Criteria

What do research panels and committees do and how do they use (and possibly abuse) bibliometric data and analysis? There is, as Jappe et al. (2018) noted, a gap between these context-specific users and the people who typically explore, analyze and propose the metrics (scientometricians).

One of us (JA) has experience of committee work at national level (as a science policy adviser in the United Kingdom and Australia) and institutional level (as Director of Research Strategy at the University of Leeds), as well as through commercial consultancy with universities in other countries. The key common learning point from these diverse experiences is that research metrics are hardly ever an arbiter in normal practice; they are more typically one of several adjunct sources of information. The information in front of a decision-making group is there to help it to arrive more confidently and speedily at that decision so as to support research management and enable activity to proceed. The presentation of a table of simplistic and opaque metrics is unlikely to do this and it competes for attention with other considerations such as apparent opportunity, real resource constraints, dominant voices, and local and third-party politics.

The United Kingdom’s Advisory Board for the Research Councils criteria for scientific priorities (ABRC, 1987) were published as a guide for both Research Council peer reviewers and committees, as well as a general aid to research planning. They draw implicitly on the ideas of Weinberg (1963) and set out criteria, both internal and external for any research project, that have stood the test of time (ABRC, 1987).

A. Internal: i) timeliness - expectation of rapid scientific advance (in 5,10 or 20 years); ii) pervasiveness - likelihood of a wide range of links with other research; iii) excellence.
B. External: i) exploitability - potential for nationally profitable industrial or commercial use (in 5, 10 or 20 years); ii) applicability - potential for uses leading to other benefits: social, environmental or related to Government policy (in 5, 10 or 20 years); iii) significance for education and training.

The ABRC noted that in all judgements, whether internal or external considerations are to the fore, the question of affordability comes into play: the likely benefits of research programmes (as for any other form of public expenditure) must always be weighed against their cost.

Excellence is one among six ABRC criteria and the only one where bibliometric data appear likely to support decision-making more effectively (see Bornmann, 2014). We will show later in this paper that bibliometrics can in fact also throw light on timeliness and pervasiveness. Moed (2005, page 57) also makes the point that citations discriminate best between good and bad but less well between good and excellent. Context, reflected here in the external criteria, is always an essential part of evaluation and Nature (2018) drew attention to the truism that “Excellence depends on context.” What is excellent in advancing basic knowledge may not address immediate problems, and vice versa.

These criteria provide a balance of reference points for a working framework (sensu Moed, 2020), which is a fundamental requirement for evaluation. Defining context and purpose provides a framework, or scenario, in which bibliometric analysis is introduced as a purposive tool, almost certainly to improve broader interpretation and understanding, increase confidence in the overall information pool through challenging heuristic assumptions (Bornmann and Marewski, 2019) and thus inspire greater and more rapid progress toward a decision.

A structure for consideration of the context for ‘good research’ has been proposed by Langfeldt et al. (2020) and they discuss three perspectives from which differences of opinion may arise: 1) research fields vs. policy spaces; 2) ‘attributes’ of originality/novelty, plausibility/reliability, and value or utility; and 3) ‘sites’ where quality notions emerge: researchers, communities, organizations, funders and national policy. We agree that it would be valuable to consider how any research project or program would be seen in these perspectives before deciding how best to evaluate the work.

Bibliometric analysis without a clear locus in a contextual and evaluation framework is unlikely to be used effectively. A table of point metrics, for example, has little contextual value since it is unconnected to other aspects of the activity under review. We need instead to move to more complex perspectives, based on multiple points of reference, that explain the purpose, and hence the purposive structure, of the evaluation and enable informed interpretation and comprehension of meaning.

### An Example

To illustrate the problem of interpretation that comes from inappropriately simplistic bibliometric information, we start from a table of point metrics, consider what these would show us and then move to other analyses that may reveal alternative or nuanced interpretations. We start with bibliometric indicators for a cross-section of ten countries. Five of these might be considered to have both large and well-funded research economies (United States, China, United Kingdom, Germany and Australia) and the other five, while improving, presently have both relatively weaker funding and smaller research output (Table 1).

**TABLE 1:** Summary metrics for the research production (numbers of documents indexed in the *Web of Science*) and performance (category normalized citation impact, CNCI world average = 1.0) of a global spread of ten countries during a recent ten-year period (2010–2019). Countries are ranked on CNCI.

	*Web of Science* documents	Average CNCI	Times cited	% Docs cited
United Kingdom	1,981,903	1.41	26,932,154	65.6
Australia	888,127	1.41	12,626,406	72.4
United States	6,838,175	1.31	90,031,964	63.9
Sri Lanka	13,068	1.31	170,284	63.6
Germany	1,615,968	1.30	23,029,125	71.1
Bulgaria	38,366	1.01	360,385	60.2
China	3,743,888	0.99	39,306,476	71.5
Argentina	121,077	0.96	1,321,844	71.4
Iran	362,748	0.91	3,428,680	77.9
Indonesia	85,885	0.81	342,576	39.1

Data summaries similar to that in Table 1 can be found in many reports from government agencies and in news media. It will be immediately obvious that it tells us nothing about the subject spread of research, which would be important for any informative analysis, nor about the context of relative research expenditure, human capacity and industrial R&D of any of these countries.

More significantly, from the perspective of the present paper, we see results that are at least likely to raise eyebrows if not actually to induce skepticism about the data source. Does Sri Lanka really have an average CNCI equal to the United States when the latter produces more than 500 times as many publications? What does it mean if Iran has the highest rate of cited papers when it is the second lowest in average CNCI? How, in other words, are these point metrics compiled and calculated?

**Figure 1:**
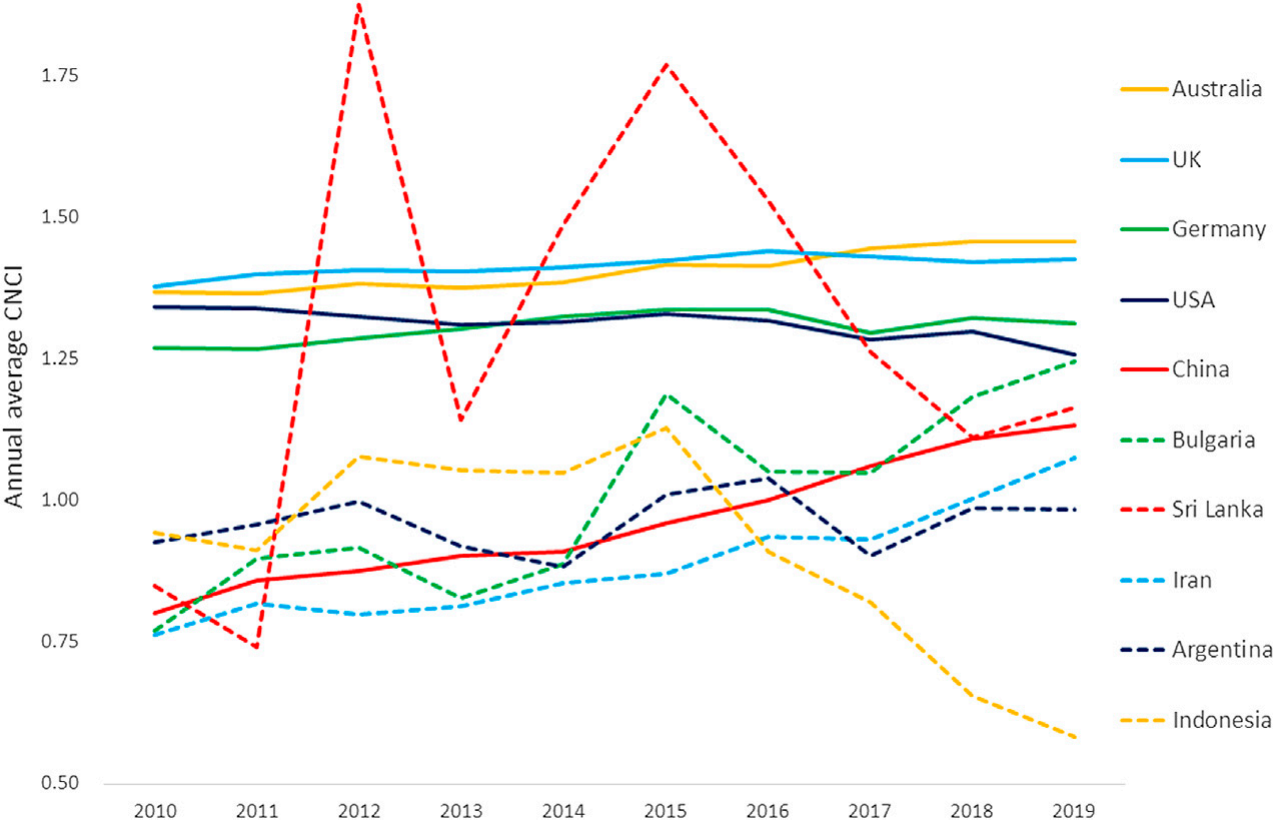
Annual trends over the last decade in national Category Normalized Citation Impact (CNCI) for the ten countries summarized in Table 1.

We can also question the representative nature of ‘average’ or total values of activity across the period. Annual trends in CNCI for the large, well funded research economies appear to be fairly steady across the decade, improving in three cases albeit drawing attention to a gradual decline for the United States. China has a steady upward trend in impact, and Bulgaria also improves throughout though its smaller output means that its line is more variable. Sri Lanka dives, however, from an exceptional CNCI in 2015 and Indonesia falls from slightly above world average to barely 0.5 of that benchmark in 2019 (Figure 1). Evidently factors other than the innate research competence of the economy are at work in these instances of indicator volatility. These both are small research economies, relatively low in their research investment and–as we shall see–highly engaged in international research collaboration.

What do the numbers tell us? The data suggest that average CNCI for at least two of the ten nations is unreliable, since doubts about the relative average for Sri Lanka in Table 1 seem confirmed by its volatility. Does that introduce doubt about the more stable values? It certainly raises questions about the detail in the evident mass of publications (Table 1) that feed the indicators for the larger economies. How representative can a single indicator be when it is chosen to stand for millions of publications and tens of millions of citations? More information is required to properly interpret either a table or a graph of summary metrics. Relevant factors explored over the last 2 decades include data granularity, collaboration, geography, history, national research culture, and accessible visualization of underlying distributions that reveal the broader context of the research under evaluation.

## Reinterpretation

### Granularity and Categorization

The CNCI values shown in Table 1 and tracked in Figure 1 are calculated at the level of the journal-based categories used in the *Web of Science* (WoS) of which the 254 current categories cover all subject domains in the sciences and arts. Separately, Clarivate also has an Essential Science Indicators (ESI) classification with 22 broad categories that do not include arts and humanities. The Clarivate InCites platform offers additional options to users, including the popular Australia New Zealand Standard Research Classification system (https://www.arc.gov.au/grants/grant-application/classification-codes-rfcd-seo-and-anzsic-codes) which is a hierarchy of Fields of Research (FoRs) with 22 FoRs at the highest level and then nested fields at increasing granularity, thus: Division 03 Chemical Sciences; Group 0302 Inorganic chemistry; Field 030206 Solid state chemistry. InCites has other classifications such as those used in Brazil by CAPES (Coordenadoria de Aperfeiçoamento de Pessoal de Nível Superior) and FAPESP (Fundação de Amparo à Pesquisa do Estado de São Paulo) and some developed for particular purposes, such as the RAE/REF Units of Assessment (UoAs) used in the United Kingdom. All of these have validity and utility in their relevant context, none are either right or wrong, but it is important that users understand which classification they have applied and what its purpose and properties may be.

For example, in calculating CNCI, the citation count for a specific publication is compared to (i.e. normalized against) the world average for the year of publication of all documents of the same type (such as article, review or conference proceeding) in the same Web of Science category as the journal in which the publication appeared. Zitt et al. (2005) drew attention to the possibility that CNCI would change according to the level (described as the ‘zoom’) at which any normalization occurs. The possible effects of changing the reference point at which normalization is made had also been noted by Hirst (1978) in relation to ‘Discipline Impact Factors’; methods for comparing bibliometric indicators across fields have been reviewed by Schubert and Braun (1993, 1996); and Glanzel and Moed (2002) commented on the effect of different levels of aggregation.

To explore how the categorization of the data might influence the type of metrics in Table 1, we tested the effect of the ‘Zitt zoom’ on our perspective of research performance by analyzing the relative impact of articles submitted for assessment in the United Kingdom RAE2001. We compared impact at three different levels of normalization for university departments at the three highest grades (4, 5 and 5*) awarded in three Units of Assessment (UoA13 Psychology, UoA14 Biological Sciences and UoA19 Physics). The outcome was a significant positive correlation between peer judgements and citation impact at some, but not all, levels of data aggregation.

The citation count for each paper was individually normalized against the average counts–taking note of publication year - for the journal in which it was published, for the WoS category to which the journal was allocated and for the complete data pool for the relevant UoA. When citation counts were normalized at journal level there was little evident difference between performance at any grade, so no link could be made between peer review outcomes and a citation index. But when the normalization was relative to the WoS category or the entire UoA, then on average the higher graded units had a statistically significant higher relative impact. These data support Zitt et al.’s (2005) analysis (Table 2).

**TABLE 2:** The average Category Normalized Citation Impact (CNCI) of articles and reviews published during 1996–2000 by research staff at United Kingdom universities for units graded 4, 5 or 5* in the Research Assessment Exercise 2001 (RAE2001). Data are shown for three Units of Assessment (UoA) with the numbers of units at each grade and the CNCI for their publications with citation counts normalized at three levels of granularity: the journal of publication; the *Web of Science* (WoS) journal category; and the data set for the entire UoA (Adams et al., 2008).

Grade at RAE2001	UoA13 psychology				UoA14 biological sciences				UoA19 physics			
	Average CNCI				Average CNCI				Average CNCI			
	Number of units	Journal based	WoS based	UoA based	Number of units	Journal based	WoS based	UoA based	Number of units	Journal based	WoS based	UoA based
Grade 4	17	1.22	1.40	0.80	17	1.29	2.35	1.89	15	1.28	1.84	1.98
Grade 5	17	1.18	1.80	1.05	30	1.11	2.33	2.33	23	1.47	2.51	2.96
Grade 5*	12	1.32	2.38	1.63	11	1.18	2.53	2.93	5	1.82	3.32	3.75

This has practical implications for research evaluation. The implication is that the material submitted by units that peer reviewers graded at 4 is actually sourced from journals of lower average impact than the material submitted by the units graded at 5 and 5*. Thus, when the level of analysis is relative to journal these items appear to be of similar impact relative to the medium in which they are published. When the viewpoint is zoomed out to the WoS categorical level then the higher absolute citation count for the articles produced by the more highly graded units becomes apparent, and even more apparent at the UoA-level.

The possibility that the level of ‘zoom’ will affect our assessment of relative impact is an important insight. A clear risk is that very fine-grained assessment becomes self-referential. Clearly, the existence of more than one view and hence more than one interpretation of performance would need to be taken into account in any evaluation methodology. Ideally, the appropriate level of ‘zoom’ would be independently considered, explored and reported before confidence in the outcome of assessment could be validated. This is likely to be a serious challenge unless a reference indicator is available and will generally require any evaluation to be carried out at multiple levels for a reflective review.

It should also be noted that not all classification systems draw on all available data. The ANZ Fields of Research (FoRs), for example, are used in the ‘Excellence in Research for Australia (ERA)’ evaluation process where submissions made by universities are assigned to FoRs by reference to expert-assigned journal lists. This results in a marked reduction in the volume of articles and reviews compared with the numbers indexed for any country or institution within the Web of Science. Table 3 shows the ratio between the total available publication dataset and the number actually assigned to each country via six other schema. Some schema, especially the journal lists for the ANZSRC Fields of Research, reduce the available data for countries such as Indonesia by as much as half. Even for the United States and the United Kingdom the publication set is down by 20% (the broad L1 categories) or 35% (the specific L2 categories). By contrast, the schema for the United Kingdom’s REF and those used in Brazil by CAPES and FAPESP essentially draw on the full source material.

**TABLE 3:** The ratio between numbers of papers assigned to the ten countries listed in Table 1 via the Web of Science journal-based disciplinary category scheme and six other categorical schema used in Clarivate InCites (schema identified in Note). The variations in the proportion of the literature that is covered will affect both the numerator and denominator citation counts in any subsequent normalization calculation of citation impact (see Figure 3).

	ESI	For L1	For L2	REF2014	CAPES49	FAPESP
United States	0.85	0.80	0.64	1.00	1.00	1.00
China	0.78	0.71	0.54	1.00	1.00	1.00
United Kingdom	0.81	0.80	0.65	1.00	1.00	1.00
Germany	0.84	0.77	0.61	1.00	1.00	1.00
Australia	0.86	0.84	0.67	1.00	1.00	1.00
Iran	0.90	0.80	0.64	1.00	1.00	1.00
Argentina	0.90	0.82	0.66	1.00	1.00	1.00
Indonesia	0.34	0.35	0.26	0.98	1.00	1.00
Bulgaria	0.75	0.60	0.48	0.98	1.00	1.00
Sri Lanka	0.76	0.72	0.59	0.99	1.00	1.00

**Note:** (ESI = 22 Essential Science Indicators journal categories excluding Arts and Humanities; FOR = ANZSRC Fields of Research where L1 = journals mapped to 24 broad categories and L2 is 212 specific categories nested within L1; REF2014 = 35 of 36 United Kingdom subject panels for Research Assessment Exercise 2014; CAPES = a Brazil schema of 49 evaluation areas used by Coordenadoria de Aperfeiçoamento de Pessoal de Nível Superior; FAPESP = 72 categories used by Fundação de Amparo à Pesquisa do Estado de São Paulo, Brazil; PL19 = the Polish schema of 44 categories used for a 2019 evaluation exercise).

Each scheme has been designed with a particular purpose in mind and draws on and organizes the literature accordingly. The variation in dataset coverage is an intentional outcome of this. However, should the unwary employ a scheme that ‘looks right’ without recognizing its characteristics then they will obtain a result that may differ from their expectations (Table 3).

Categorical schema also have an effect on CNCI, as seen in the ‘Zitt zoom’ example in Table 2. Unsurprisingly, indeed reassuringly, there is a very high degree of correlation between the CNCI values obtained from citation counts normalized under different categorical systems. However, the correlation is not perfect and there can be differences both in the *y*-intercept, which would move all values up or down, and the slope, which would differentially affect organizations with lower and higher average impact. Matching data categorization to the objectives of the assessment is therefore essential if equity is to be maintained across all parties under assessment.

**Figure 2:**
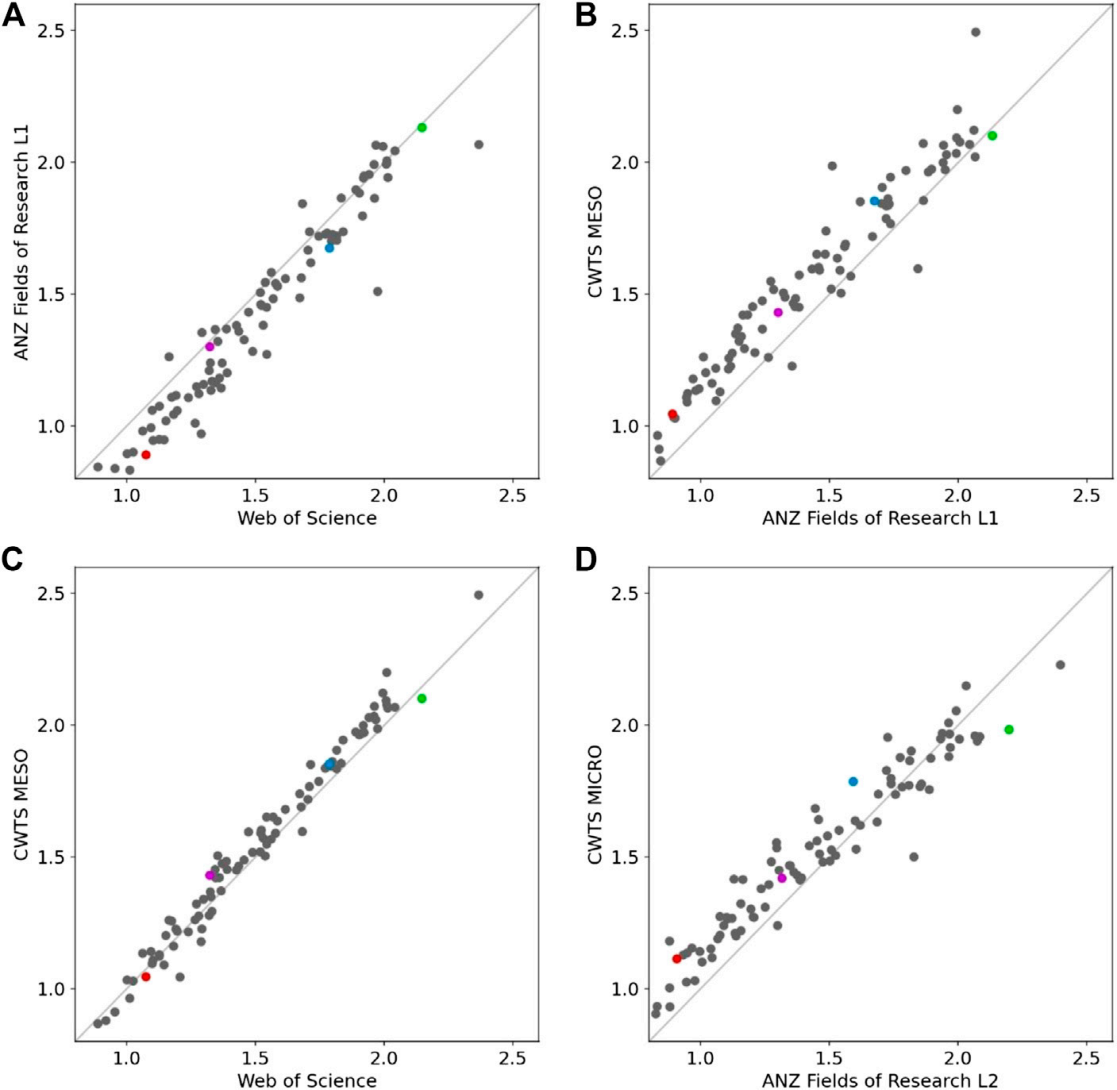
**(A)–(D)** Correlations between the average Category Normalized Citation Impact (CNCI) of United Kingdom universities (*Web of Science* indexed publications for 2015–2019) when different schema (see Note) are used to categorize the institutional and global publication data. In each case the correlation is highly significant but the variance about the regression differs for specific institutions. Four universities with distinct research histories and portfolios are highlighted with a constant color point. (*Web of Science* (WoS) categories map journals to 254 fields; ANZSRC Fields of Research (FOR) use L1 = journals mapped to 24 broad categories and L2 = 212 specific categories nested within L1; CWTS MESO and MICRO refer to coarse and fine citation-based categories developed by CWTS, Univ of Leiden). **(A)** FOR1 vs. WoS, *n* = 86, correlation = 0.968. **(B)** CWTS Meso vs. FOR1, *n* = 86, correlation = 0.954. **(C)** CWTS Meso vs. WoS, *n* = 86, correlation = 0.986 **(D)** CWTS Micro vs. FOR2, *n* = 86, correlation = 0.926.

The average CNCI for all United Kingdom universities (2015–19), taken across all discipline categories in each of several different categorical systems, is shown in Figure 2. The effect of moving from WoS journal categories to the FOR 2-digit Level 1 is to depress most institutional CNCIs but this effect is most marked below world average CNCI and almost negligible at the upper end of the distribution. There are also some evident outliers, so the effect is far from uniform. There is a much closer correlation between the CNCI values for the WoS categories and the topical categories created by a citation-based clustering developed by the Center for Science and Technology Studies (CWTS, University of Leiden). Specifically, we used the ‘meso’ level in CWTS’s three tier system. Comparison between the CNCI outcomes using CWTS meso categories and the FOR1 categories shows again that the FOR system depresses the CNCI values. A shift to a finer-grained level, using the CWTS micro and the ANZ FOR Level 2 categories, produces a similar effect but the change in slope is more evident and the depression in the low CNCI part of the distribution is relatively greater (Figure 2).

The changes in relative positions for the four tracked universities illustrates the considerable residual variance in these example graphs. The shift from one categorical system to another is never uniform across all the entities. Comparing WoS with FOR1 (Figure 2A), there are six universities with an average CNCI of 1.7 when using WoS journal categories that would achieve CNCI values ranging between 1.45 and 1.85 if FOR1 categories were used for data grouping and normalization. Looking at the four tracked universities in comparisons between CWTS-MESO and FOR1 (Figure 2B) and between CWTS-MICRO and FOR2 (Figure 2D), the highest performer university gains in the shift to FOR but the other three all suffer a reduced CNCI.

These shifts may be due to subject mix, because each system assigns journals differently across the specific category series so the content of global baselines changes, or it may be another, less apparent factor, but it materially affects the relative institutional outcomes and cannot be ignored.

**Figure 3:**
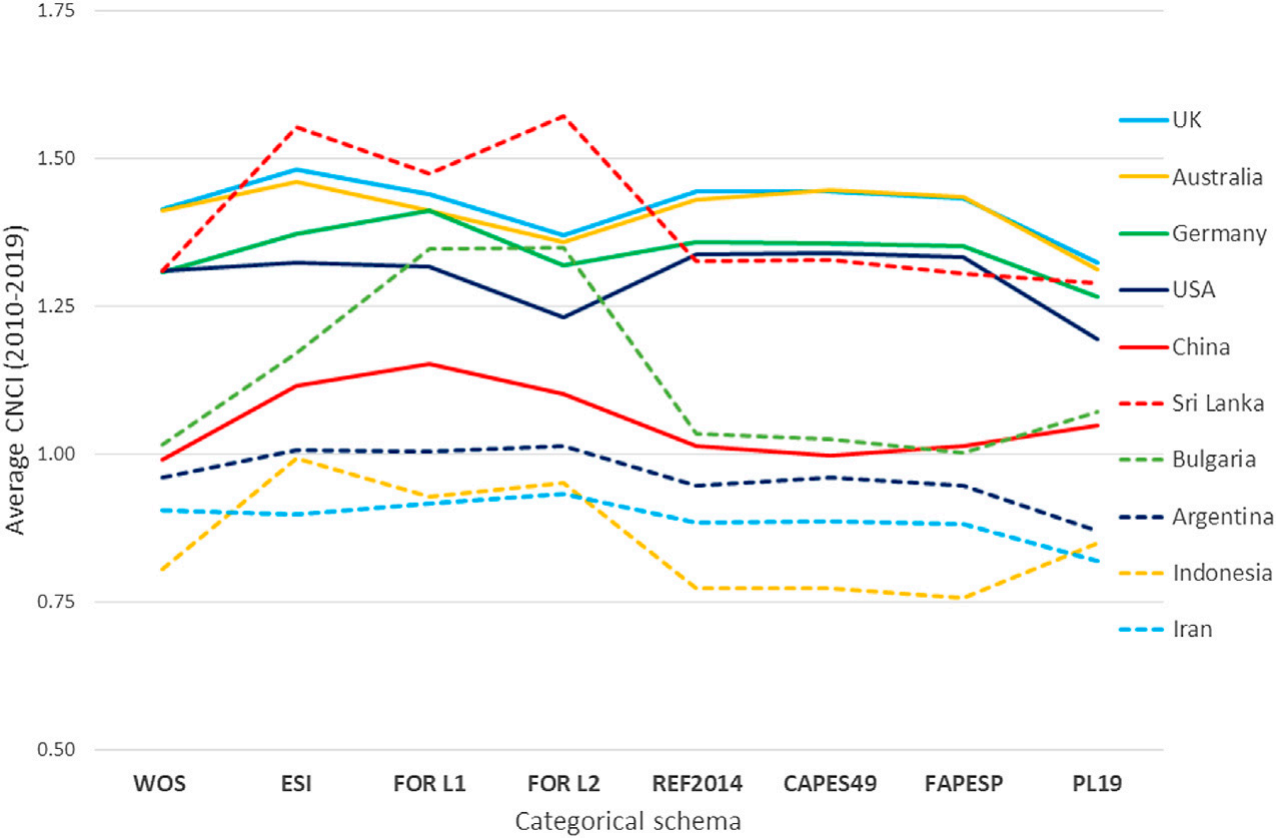
The average Category Normalized Citation Impact (CNCI) for ten countries calculated with data normalized under eight different categorical schema. The numbers of publications used to calculate CNCI vary between schema as indicated in Table 3. The graph lines do not imply any connection between distinct schema but are inserted as a visual aid.

The effect of this on the ten countries in Table 1 reflects these trends and is, in some instances, noticeable (Figure 3). The data in Table 1 (based on WoS journal categories) suggested that CNCI for Sri Lanka was similar to that of the United States and Germany. The use of the ESI schema or either of the ANZ FoR schema produces an outcome in which Sri Lanka is apparently world-beating. Indonesia’s CNCI is also elevated if these schema are used, but the CNCI of most countries is generally affected much less although that of the United States, United Kingdom, Australia and Germany are all slightly depressed under FoR Level 2 and the Polish PL-19 schema. Indonesia benefits under the Polish schema but Sri Lanka does not.

They key lesson here is that the way in which the data are selected and aggregated will have an influence on analysis and interpretation, yet none of these alternative schema have been implemented casually or without planning, analysis and prior development.

### Collaboration

The global research landscape has changed considerably over the last forty years. In the 1980s it was dominated by a *trans*-Atlantic axis with links to Japan and to Anglophone countries with established university systems on the European model. In 2020, the balance of the research world has changed: Asia-Pacific plays a key role, through China (the second largest research economy in Table 1), South Korea, Singapore and a network that stretches to Australia (higher CNCI than the United Kingdom or United States in Figure 1); there is another, growing network across the Middle East and North Africa; and Latin America waxes and wanes as economic cycles create opportunity.

**Figure 4:**
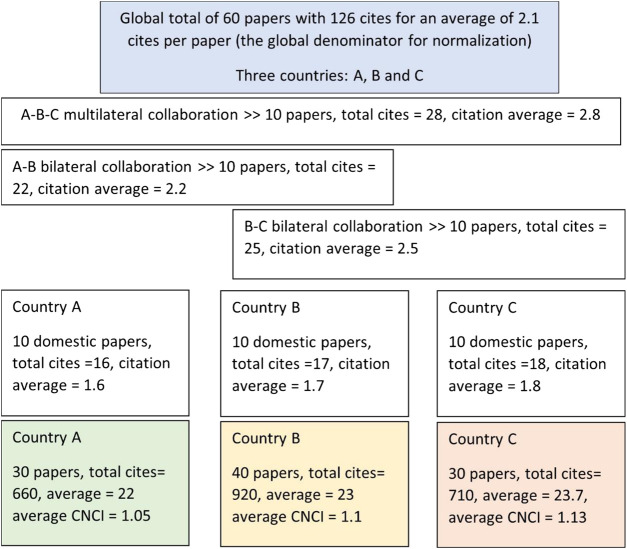
**(A)–(D)** Impact Profiles for four G20 countries for articles and reviews indexed on the *Web of Science* during the ten-year period 2009–2018. Each profile includes three extracts for the country plus a reference benchmark taken from the complete G20 dataset. The three extracts for each country are the Impact Profile curves for: total national output; domestic output (with no international co-author); and internationally collaborative output. Each curve shows uncited papers (histograms to the left) and the distribution of output across eight categories of increasing impact relative to world average. The green line is a common reference set for all the graphs and marks the average for the complete G20 dataset. **(A)** China **(B)** Germany. **(C)** Argentina **(D)** Indonesia.

There has been an increasing level of international collaboration across this dynamic world network (Georghiou, 1998; Wagner and Leydesdorff, 2005, Wagner, 2008; Leydesdorff and Wagner, 2008). International collaboration has generally been seen in policy research discussion as a supportive research strategy enabling access to greater intellectual and economic resources and accelerating work both on researcher-driven projects and on strategic programs such as those in particle physics and on the human genome. For this reason, it is often monitored and promoted as part of national research policy (for example, in EU policy and the EU’s Horizon 2020 research program (https://ec.europa.eu/research/iscp/index.cfm?pg=policy). It is also associated with increasing citation impact (Persson et al., 2004) and internationally collaborative papers are more frequently cited on average (see Figure 4 later).

**Figure 5:**
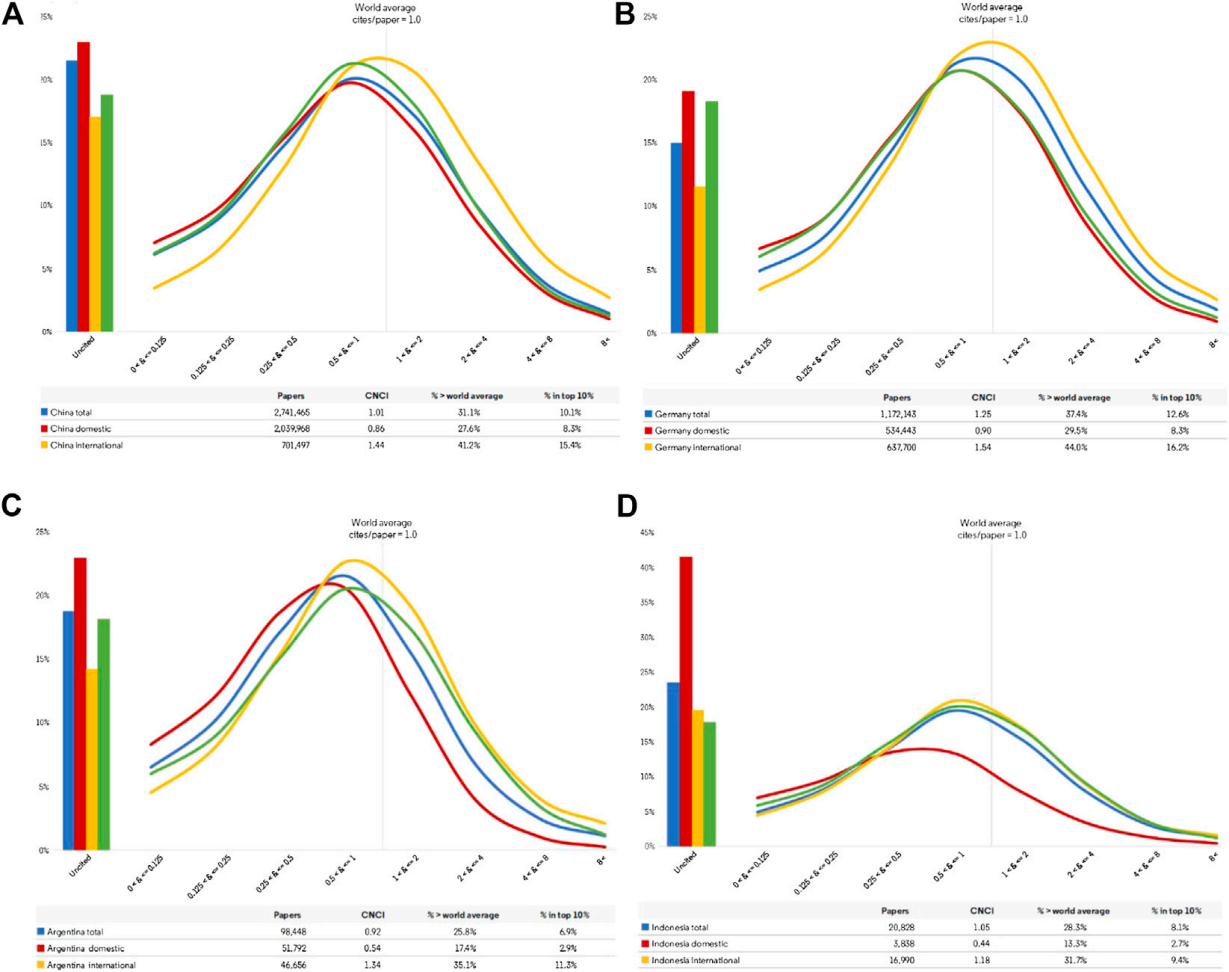
**(A)–(D)** Output indexed on the *Web of Science* for Australia, Germany, Indonesia and Iran deconstructed by total and purely domestic articles and reviews. The domestic share of output has steadily declined for the large research economies while output, boosted by collaboration, has steadily risen. Output for the smaller economies has risen more steeply but the profile of international collaboration is less consistent. **(A)** Australia **(B)** Germany. **(C)** Indonesia **(D)** Iran.

Analyses by ISI (Adams, 2012; Adams, 2013) over the last ten years have identified changes consequent upon these increases in international collaboration that alter the structure of the national research base. For Australia and Germany, as examples of large research economies, international collaboration has in fact become the critical driver of rising productivity (Figures 5A,B). Almost all increase in annual publication counts can be accounted for by output shared with one or more collaborating countries whereas the domestic research output (with no international co-authors) has plateaued.

The pattern for countries that are still growing and developing their research economies may be quite different. Indonesia’s overall output has risen steeply but its level of international collaboration has always been very high and has increased so that a very high proportion of its output over the last decade has been collaborative (Figure 5C). Iran also has steeply rising research output but it is almost entirely driven by the domestic research base and its international collaboration has been much lower (Figure 5D).

The United Kingdom and Germany share around 10% of their output with one another and each shares around two-thirds of its annual output with other countries. This pattern is similar across the European Research Area and mirrored by most other advanced economies. The internationally collaborative part of each country’s output is also the more highly cited (Adams, 2013), which is unsurprizing since collaboration requires a shared agenda: a compromise that must be offset by clear likelihood of research benefit.

The innate, historical research strength of the larger, established economies countries means that while collaboration may boost their performance as measured by average CNCI it does not alter it disproportionately. However, the contribution made by different partners is not uniform. Adams and Gurney (2018) showed that the United Kingdom ‘gained’ in citation impact when collaborating with the United States, Germany and France and the average CNCI of such papers was as much as twice world average. This citation boost changed when, instead of all co-authored papers irrespective of third parties, only bilateral papers were considered. The United Kingdom still gained but for German and French collaborations it did so only marginally. This separation of bilateral and multilateral components may become increasingly important (see also Table 4).

**TABLE 4:** Total national papers and those co-authored between a European country and former colony (2015–2019). Collaborative papers may have other, third-party countries as co-authors so both the total collaborative and the solely bilateral counts are shown.

Collaboration: All/bilateral		France	Netherlands	Spain	United Kingdom
	National total	398,747	221,375	321,566	666,166
Argentina	49,997	3,743/878	1,883/102	5,789/2,190	3,418/312
Indonesia	15,333	932/196	1,476/574	366/26	1,654/288
Kenya	10,842	720/64	890/120	400/11	2,783/521
Tunisia	23,013	6,973/4,670	186/9	1,547/795	596/87

**Figure 6:**
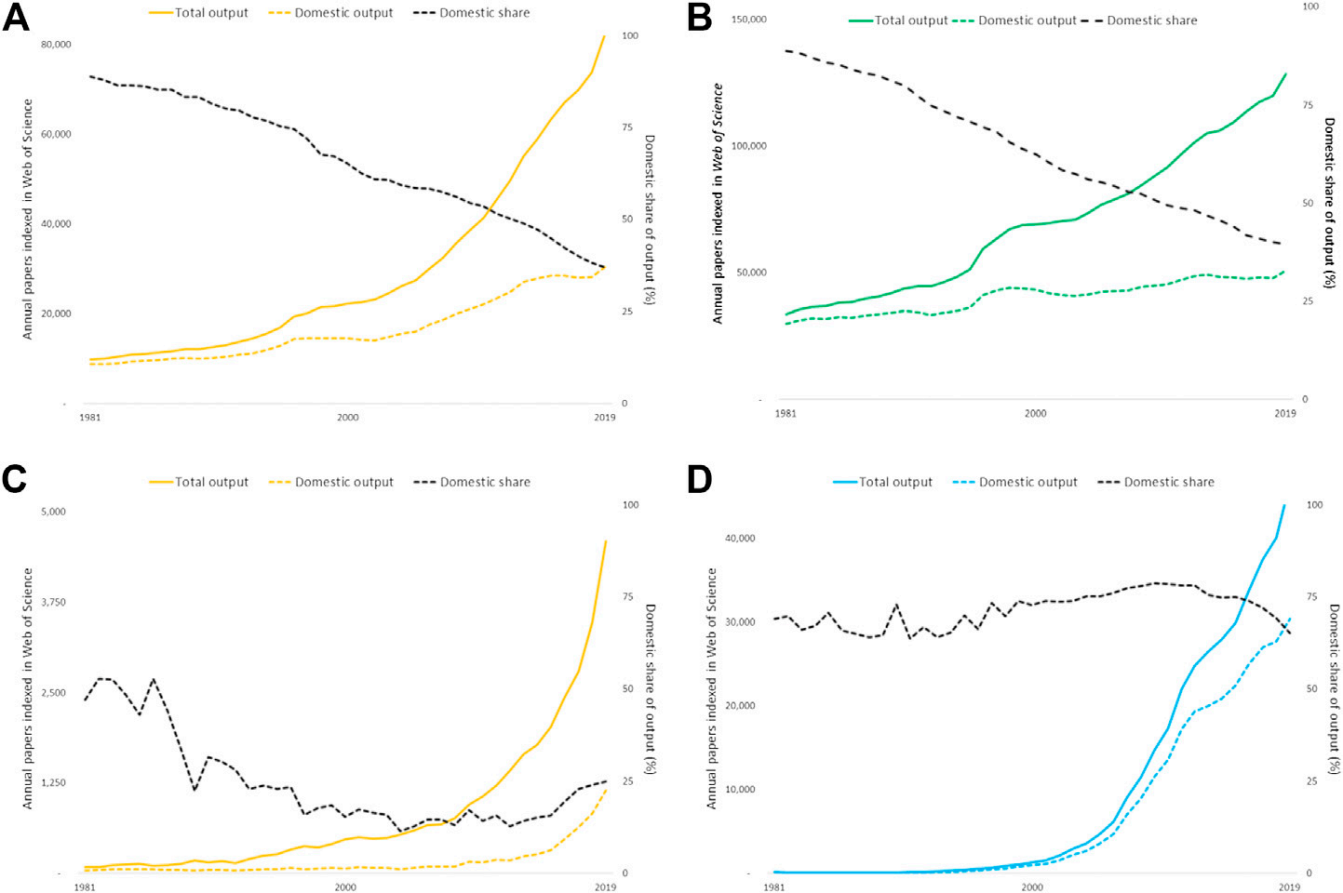
**(A)–(D)** Deconstructed article and citation distribution output for China, the United Kingdom, Sri Lanka and Indonesia (2009–2018). Articles are divided into five types: domestic single (dom_single) and multi (dom_multi), and international bilateral (int_bi), trilateral (int_tri) and quadrilateral-plus (int_quad+). White squares on boxplots represent the mean. Data reproduced from Potter et al. (2020, Figure 6). **(A)** China **(B)** United Kingdom. **(C)** Indonesia **(D)** Sri Lanka.

Disproportionate change due to collaboration can compromise the research metrics of smaller economies such as Indonesia with a shorter history of investment and growth. We analyzed the parts of national output that are accounted for by domestic authorship (both single and multiple), bilateral international collaborations, trilateral and multilateral collaborations. We counted the numbers of articles and reviews produced over the decade from 2009 to 2018 and calculated the share of total citations attributable to each country that were contributed by these different groups of papers (Potter et al., 2020). (Figure 6)

Domestic output for China is 75% and for the United Kingdom is 44% of total published output. This accounts for 65% of China’s citations and 35% of the United Kingdom’s (Figures 6A,B). Domestic output therefore collects *pro rata* a similar but smaller proportion of citations than it represents as a proportion of publications. However, domestic output for Indonesia (51.7% of total output; Figure 6C) and Sri Lanka (38%; Figure 6D) accounts for a much smaller share of national citations received (around 10%). By contrast, their highly multilateral papers (respectively 7.3% and 15.3%) account for, respectively, 36.4% and 59.8% of the citations they received (Figure 3) and, thus the overall CNCI figure is highly dependent on the performance of the multilateral collaborations to which they contribute. By contrast, highly multilateral papers were 1.6% of output and 4.4% of citations for China and 10.2% of output and 21.8% of citations for the United Kingdom. In other words, the large research economies not only gain relatively more citations from domestic output but while their multilateral collaborations certainly augment overall performance metrics they do so to a lesser extent: by a factor of 2 rather than four to five.

Referring back to Table 1, we conclude that the exceptional average CNCI for Sri Lanka appears to be dependent primarily on its collaborations rather than its innate research profile (Figure 6D) whereas that of China is clearly proportionate to the balance of domestic and collaborative activity (Figure 6A). The particular annual values are dependent on the numbers of such collaborative papers in that year and the time they have had to receive international recognition whereas the more stable CNCI metrics for the large economies are attributable to innate national research activity and recognition. The implication is that it is not sufficient to evaluate national bibliometric performance solely through summary indicators but it is essential to understand the balance and stability of domestic, collaborative and highly collaborative activity that feeds into such indicators and to be aware of which other countries may be involved in such collaboration (see also Table 4).

### Fractional Attribution

It has historically been the practice to assign the full value (of both production credit and CNCI value) of a publication to each author, each institution and each country listed in the author metadata. This may cover participation but it does not necessarily reflect contribution. Given the collaborative nature of research, it has been argued that fair assignment of credit to the authors is not only important but essential (Allen et al., 2014) and this perspective is increasingly supported by the academic community because of its significance for funding (Sivertsen, 2016), promotion (Klein and Falk-Krzesinski, 2017), and national standing (Ahmadpoor and Jones, 2019). However, Larivière et al. (2020) both argue that the interpretation of contribution roles may vary as widely as criteria for authorship in different disciplines and that attribution of leadership and supporting roles may become a divisive and value-driven process.

One frequently proposed alternative is fractional counting (Waltman and van Eck, 2015) whereby each author is assigned part of the credit and CNCI value. From an aggregate perspective, fractional counts add up to the same number of articles as are in the data, which may provide better balance and consistency in bibliometric indicators but it is also claimed to improve precision: an assertion that is unprovable and misleading. Equal is not the same thing as equitable in the distribution of credit, and this is evident among international multilateral papers (Figure 6).

An even fraction may accurately reflect credit for some small groups (perhaps up to four individual entities?) but no algorithm will allocate credit proportionately among larger groups where major and minor contributors must be present. Sivertsen et al. (2019) showed that median authorship rates vary markedly between fields. They proposed a family of indicators for modified fractional counting (MFC) based on the root of the fractional authorship, which they argue eliminates extreme differences in contributions over time that otherwise occur between scientists that mainly publish alone or in small groups and those that publish with large groups of co-authors.

Another approach is to enhance CNCI normalization. There is a clear disparity in article volume, citations and CNCI between different collaboration types and countries (Figure 6). Potter et al. (2020) proposed a new metric, ‘Collab-CNCI’, that accounts for the level of collaboration without presuming credit. Their analysis demonstrates that Collab-CNCI reduces the impact of highly collaborative articles on a country’s mean CNCI when using the full count method, providing a more balanced view than the standard mean CNCI. The relative decrease in mean CNCI was greater for the smaller research economies, where, generally, multilateral collaborations make up for a larger and sometimes disproportionate percentage of their publication output.

### History and Geography

The collaborative links for many research economies are influenced not only by their capacity, but also by their geography and history, particularly where there are significant global links to former world powers.

The United States appears to be less collaborative internationally than other G7 economies (Adams, 2013) but this may be, at least in part, a consequence of its location (with borders on the Atlantic and Pacific Oceans) and the great size of its domestic economy. It is as far, and takes as long to fly, from Los Angeles to Boston as from London to Ankara but the latter route crosses many borders in the European Research Area. New Zealand’s remote location may explain why it is less collaborative than the similarly sized Denmark: both are strong research economies but the latter is positioned in the European network.

Links to former colonial powers are also reflected in many concentrated collaborative partnerships. We can consider the relative number of collaborative papers between four large European research nations that previously occupied territories in other parts of the world. Comparison of total and purely bilateral international collaboration suggests that historical ties and language shared between Spain and Argentina make this a stand-out relationship for both countries. About 12% of Argentina’s publication output is collaborative with Spain and more than one third of those papers are purely bilateral, with no third-party participation. This compares with its collaboration with France, the United Kingdom or the Netherlands where it has fewer shared and many fewer bilateral publications. France evidently has a far stronger relationship with Tunisia and collaborates on almost one-third of that country’s publications, with a high proportion of purely bilateral co-authorships (Table 4).

The United Kingdom has strong ties to Kenya and is a co-author on about 25% of that country’s papers, many more than any other EU nation. The five-year total tally is actually fewer than that between the United Kingdom and Argentina, but the bilateral tally is not. The significance of the bilateral component is again affirmed by the links between Indonesia and the Netherlands: the larger United Kingdom has slightly more collaborative papers with Indonesia than does the Netherlands but the latter has twice the number of bilateral co-publications.

The significance of these national links is that they are an indicator of two things: a prior cultural influence that is likely to be reflected in the research structure and portfolio of the growing economy; and an overlapping component in publication and citation data. It is infeasible, for example, that Tunisia’s average CNCI is not associated to a marked degree with that of its collaborators in France.

The overall pattern of collaborative links for Africa (Adams et al., 2014) confirms the residual legacy of previous colonial links, often traceable to institutional associations through a shared European language that became the foundations for later collaborative networks. A West Africa group (Benin-Togo) pivots around Cameroon, a relatively research productive country, and the common factor within this group is almost certainly their common use of French as the cross-national business language. A large group of collaborative nations in East Africa includes Kenya and geographical neighbors but also includes West African Nigeria, Ghana and Gambia which share English as a common language.

Such bilateral connections and local networks, drawing on a history and investment beyond the global milieu, contribute positively to overall performance profiles. It is essential to be aware of such histories in interpreting and explaining activity and performance patterns for both the established and growing partners.

We also note again the effect of geography. This is immediately obvious in the East and West networks within Africa although they are also influenced by the major communication factor of shared language. In North Africa, we can see that the Mediterranean location of Tunisia has sustained its historical links to France and enable it also to have substantial collaboration with Spain.

### Culture

The calculation of CNCI draws upon our understanding that citation counts not only grow over time but do so at rates that vary by discipline. They are influenced by disciplinary cultures: at a broad level, between humanities and the natural sciences; at an intermediate level, between organismal and molecular biology; and at a fine level, between basic and applied work on the same topic.

A further factor, that is less often identified or understood, is the influence of national cultural differences, influences that appear linked to perceptions of the relative significance of domestic and international research.

English has become the lingua franca of international research and the use of other languages impacts visibility and citation potential (van Leeuwen et al., 2000; van Leeuwen et al., 2001). For example, Russia and Brazil exhibit strong preferences for the Russian and Portuguese languages, respectively, even within journals indexed in WoS. The extent to which a nation’s output appears in journals with a domestic rather than an international orientation appears also to have a subsequent effect on citation potential. Japan is an example of a nation that disproportionately publishes in the domestically oriented journals of the nation’s scientific and medical societies. Even when these titles are English-language and published by international commercial firms, their content is less seen and less cited than papers appearing in internationally oriented journals (Pendlebury, 2020).

Another example of the influence of national, and likely cultural, factors on indicators of national research performance is seen in our analyses of data comparing the CNCI trajectory of China with the US and major European economies, which brings out a further example of misunderstanding what particular constructions of the citation data are reporting.

The data in Figure 1 appear to present CNCI tracks for ten countries across a five-year period. In fact, the annual data points show the average citation count to date for the papers published in each of those years. The CNCI indicators for the papers published in 2015 are informed by five years of citation data, at both national and global benchmark level. The papers for 2019 have one year’s accumulation of citations at best and much less for those papers published later in the year.

The format of Figure 1 is typical of that in many national and agency reports, but is it a fair reflection of performance and, more specifically, of the trajectory of performance across a period? Concern about the number of analyses that appeared to suggest that China, despite its growing research investment, was failing to deliver research of quality, led ISI to an analysis that compared the picture presented by the traditional historical analysis with a different deconstruction, one that followed the performance of an annual cohort of papers as citations accumulated over time for specific countries and for the global benchmark: this presents a different perspective (Adams, 2018). The key here is that the indicator is not tracking a change in performance over time but the record of performance for different cohorts of papers based on citations recorded to date.

**Figure 7:**
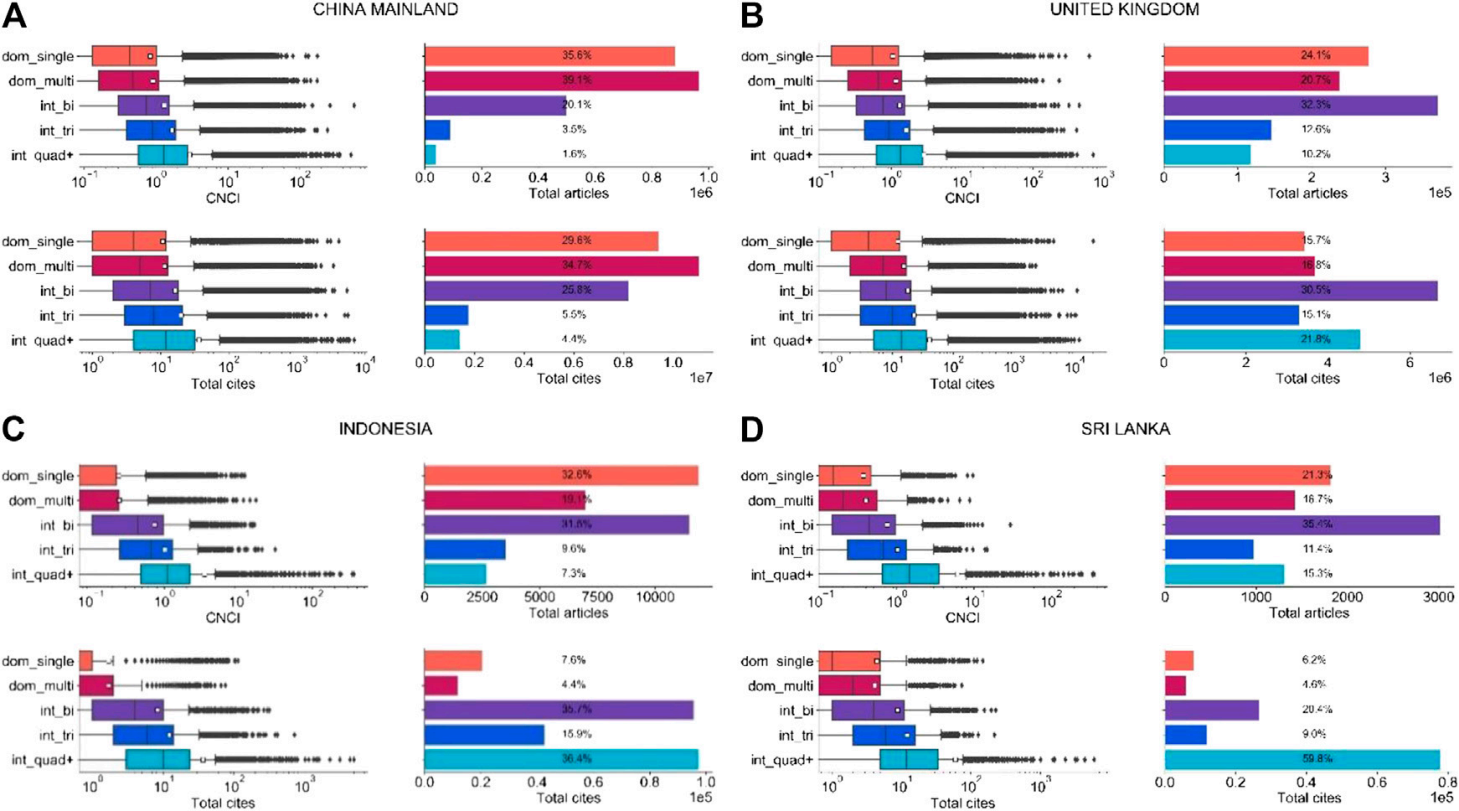
**(A)**, **(B)** Average annual CNCI for Germany and for China as captured in successive time series from National Science Indicators (NSI, using versions published annually from 2007 to 2012) based on data in *Web of Science*. Each successive NSI time series uses all available data at the census date to illustrate an implicit national trend based on citation counts to date for the publications of each year, with the publication year indicated on the *X*-axis. Comparison vertically, i.e. between the NSI values of a specific publication year, shows the real CNCI trend of any one year’s papers in successive NSI versions. **(A)** Germany **(B)** China.

In conventional analysis, analysts illustrate a performance trend using all available data, which means counting and normalizing all citations to date for the publications of each year in the series. The series then shown is not a performance track for any particular set of papers but an implied track for the entity as a whole, where normalization compares the entity to the global average. A conventional time-series analysis based on all the available data at a single census point (drawn from the National Science Indicators published annually from 1992 by ISI and then by Thomson Reuters) would, for example, suggest that Germany is on a clear upward trajectory but that while the average CNCI of China’s output is unquestionably improving, it tends to fall in relative performance in the most recent year of each series (Figure 7).

However if, instead of looking at the CNCI for publications in a series of years, we track papers from a particular year over time as citations accumulate both to our target cohort to the rest of the world’s papers published in that year, then we see that the CNCI of German papers falls in later years after a relatively high level achievement in the years immediately after publication (i.e., the 2006 papers have their highest CNCI in the 2007 series and then drop lower in each later version). Each time series in successive versions of our NSI versions essentially mimics that of the previous and there is little net improvement. By contrast, the trajectories for China progressively improve in CNCI relative to world average (i.e. the penultimate year of every series is at a successively higher CNCI value than any previous publication set).

Annual United Kingdom CNCI data follow the same pattern as Germany and the United States falls off even more markedly. Which is the ‘correct’ analysis? Neither: both are necessary for a fuller understanding of performance dynamics.

Thus, it appears to be China and not Germany which is ‘on the up’. Why should the citation impact trajectory of China’s output differ from that of the West? We cannot be certain about this but there are several possibilities. First, there may be a tendency in Western research economies to focus on ‘recency’ where the latest research garners particular attention. The publications of the most recent years are those frequently cited and the citation count plateaus rapidly after that initial burst of attention. By contrast, the rapidly expanding output of Chinese researchers may be referencing the smaller body of slightly older literature which then boosts the relative citation status for those cohorts. Thus, after five years or so the average CNCI for Chinese literature has moved up on the world average while the German, United Kingdom and US literature has dropped back. A second possibility is that Western literature retains a primacy while China is still establishing its global profile. Thus, both Chinese and Western researchers focus on the latest discoveries in Europe and America first and then only subsequently does the Chinese research base recognize its own achievements.

Tang et al. (2015) have drawn attention to a “clubbing” effect in China's recent surge in research citations. For highly cited nanotechnology papers, they found that a larger proportion of Chinese citations are from domestic institutional and national networks than is true for similar U.S. papers. This may be a cultural factor, but it may equally be an indication of the degree to which Chinese nanotechnology research, which has grown to twice the size of the US, is now more citable.

Clearly, context must be assessed as well as data. Whatever the explanation, the key effect on the interpretation of research metrics is that performance trends need careful interpretation in a full understanding of the basis on which a time series has been analyzed.

### Global Benchmarks

Another, apparently artifactual and potentially confusing outcome of the pervasive growth of collaboration is that it is possible for all countries to have a CNCI value that is above the world average and yet to have more than half their output below world average. This contextual information is rarely apparent to subject-expert evaluators and may consequently be disturbing when encountered.

The explanation is that the global total must include all the national pools of domestic papers (relatively less often cited) plus a single, deduplicated set of the shared pool of internationally collaborative papers (on average more highly cited). By contrast, each country has only its own pool of domestic papers plus its portion of the collaborative pool.

**Figure 8:**
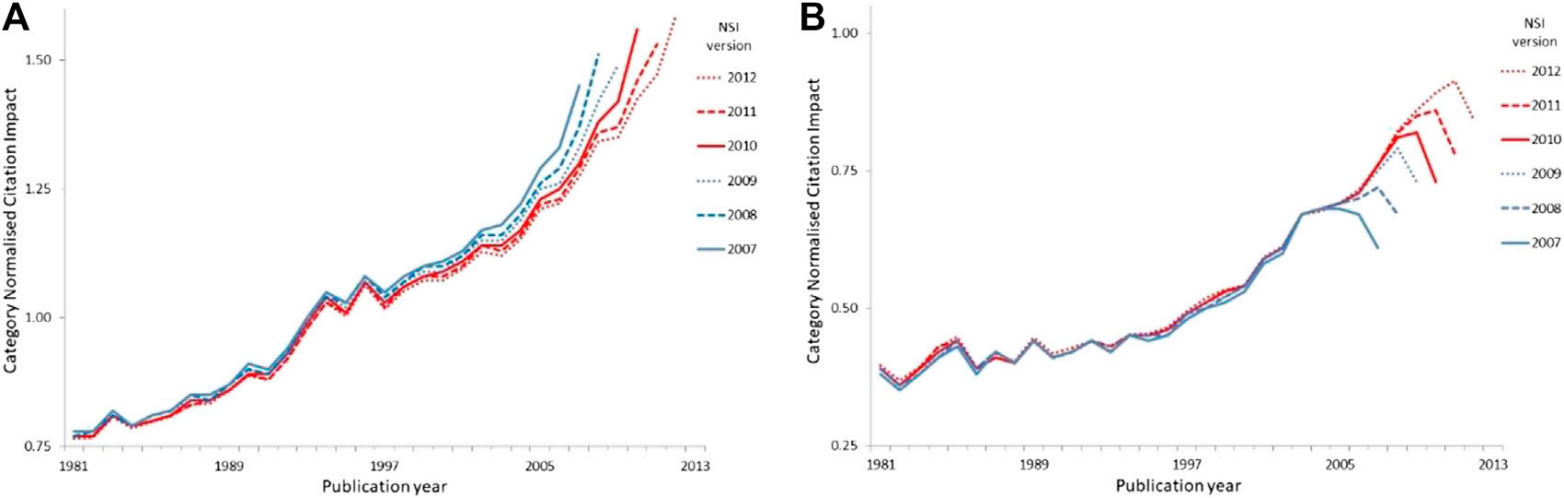
For a hypothetical universe of three small countries, with some shared and some purely domestic papers, it is evident that the global average citation count may be less than that of any one contributor. This is because domestic papers are on average less frequently cited than internationally collaborative work and the global total includes all three domestic pools whereas each country only hosts its own.

This may still seem infeasible but the schematic analysis in Figure 8 for a hypothetical world of three small countries shows that the global benchmark can indeed be below all three of the contributing nations’ individual citation averages.

It is equally the case, for a country with an average CNCI above ‘world average’, that more than half of the country’s papers will have individual CNCI below world average. The initial reaction of research managers will be that this is not possible but it is in practice not only possible but a likely consequence of the skewed nature of citation distributions that result in an average value that is well above the median. Many papers in most samples are uncited, possibly because they are recently published; most have a modest number of citations; a few will have attracted many citations. This skew is familiar to scientometricians but not to research-domain specialists and it leads us to the need for graphic illustrations of the distribution of impact that underpins the averages.

A problem arose in reality when ISI was faced with two apparently similar biomedical research units under quinquennial review which appeared to have very different performance as indicated by their average CNCI (the report on this is commercially sensitive). The solution to improved understanding, and the route to a graphical analysis that would inform and support management decision making, was to visualize the distribution of performance in ‘bins’ ranked by relative citation performance around the world average. By separating out the frequent uncited papers and then ranging the remainder in eight tranches with successive doubling of their relative impact, it is very easy to see the shape of the distribution, the balance of exceptional and weak research and to compare multiple curves or ‘Impact Profiles’ (Adams et al., 2007). In the particular instance that drove this development, it became evident that a very small number of exceptionally highly cited papers for one unit strongly skewed, even ‘distorted’, its average but the overall Impact Profiles were otherwise identical. The analysis thus validated the original views of the expert review group.

The Impact Profiles all confirm the influence on citation impact of the internationally collaborative component of each country’s activity. They show that the CNCI distribution is almost always spread across a range of impact categories from well below world average, where CNCI is 1/8th or less of world average, up through successively higher tranches. Similarly, while Germany has a high average CNCI (Table 1), it still has a substantial output of poorly cited papers, which may be a language effect (Figure 4B). No country is either completely excellent or uniformly poor in its research. Impact Profiles also enable us to introduce a reference curve, not just a single metric such as ‘world average’ but a complete profile for either the world or, as in Figure 4, a relevant reference group, which is the average for the combined G20 dataset. This also enables rapid comparison between the different countries.

An important aspect of the Impact Profile is, therefore, that it not only properly presents the distribution underpinning the CNCI indicator but it also reveals the extent to which a country (or institution or group) that has only a modest average impact may nonetheless have excellent papers in its portfolio. Furthermore, it establishes a much better contextual comparison because it does not use a single point metric as a benchmark but it can deploy a reference curve across an entire distribution. This has immediate practical applications in any research evaluation since the appearance of the higher impact papers in a profile will then prompt management questions about their authorship, the source of their citations and their links to–perhaps even dependency on - other, less prominent work. Research development and investment is facilitated by moving away from a summary to unpack the content and see a route to action.

### Context and Distributions

A shift from CNCI toward a more contextual basis for analyzing citation counts has been advocated by scientometricians (e.g., Waltman and van Eck, 2016) who have pointed to the value of percentiles as a tool for moderating both skew and kurtosis in citation distributions. The latter means that in some low-citing fields it would be exceptional to have a paper that was much above four times world average whereas in fields of citation abundance the greater spread of counts facilitates values more than eight times world average.

Bornmann et al. (2012) point to the use of a percentiles as an improved basis for an indicator of excellence in world rankings and Bornmann (2013) highlighted their analytical use in research evaluation, enabling both an assessment of the distribution of percentiles across a set and a focus on the publications with the highest citation impact. Waltman et al. (2012) discuss possible statistical problems in ranking caused by the discrete nature of citation distributions, especially with small samples, and applied a fractional solution. Bornmann and Williams (2020) discuss this and elaborated on earlier work to describe guidelines and procedures for the normalization of percentile ranks based on cumulative frequencies in percentages. They also show how graphical visualization can present this information in a more meaningful and accessible manner.

Although we have encountered an interpretive problem, in that percentiles suffer from a lack of intuitive understanding among casual users, and they may also be unsatisfactory with small samples, we nonetheless agree that percentiles generally provide a better explanatory context than CNCI for understanding the impact of a paper in its field. We note, for example, the methodology used in the Leiden Ranking of world universities (https://www.leidenranking.com/ranking/2020/list). This ranking draws on percentiles rather than normalized citation counts and applies a threshold at the top 10% of papers by field, ranking institutions according to the overall proportion of their papers that pass such a field threshold (Waltman et al., 2012).

### Context and Maps

We noted at the outset that contemporary bibliometrics can go further and address other contextual criteria set out by the ABRC (1987) including timeliness and pervasiveness. While percentiles clarify relative excellence, they do not increase the evaluators’ understanding of significance in other contexts. To do this it is necessary to determine whether the research under evaluation is part of current and substantive developments in its field, or in associated fields where it has application. Is it a part of a research cluster that is currently well-cited (timely) and is that cluster significant in scale and reach (pervasive)?

In developing the Science Citation Index, Garfield (1955) recognized that citation data provide material to build a picture of the structure of scientific research and sketch its terrain. In the previous section we arrived at Impact Profiles, which enable us to see the distribution of excellence in any dataset and then set that against a reference curve that lifts our appreciation of context beyond a point metric such as world average. This is a statistical relationship. In addition, once an index linking papers through their citations exists, there is a basis for determining their intellectual relationships. Derek de Solla Price (1965) noted, “The pattern of bibliographic references indicates the nature of the scientific research front.” This pattern provides a map in which a research publication can be located and from this the analyst can apply a time axis that shows the direction of intellectual travel. It is possible to determine where a topic is and what direction the research around that topic is taking.

Small (1973) laid the foundations for defining specialties in research fronts using co-citation analysis. Small and Griffith (1974) and Griffith et al. (1974) showed that individual research fronts could be measured for their similarity with one another and thus form the nucleus of a specialty. Their mapping used multidimensional scaling and similarity was plotted as proximity in two dimensions. There are now many academic centers across the globe focusing on science mapping, using a wide variety of techniques and tools (Börner, 2010; Boyack and Klavans, 2010; Petrovich, 2020). These later developments are summarized in Indiana University Professor Katy Börner’s (2010) *Atlas of Science*. Of particular significance are CiteSpace developed by Chen (2006) and VOSviewer developed by Van Eck and Waltman (2010) at CWTS, Leiden University.

**Figure 9:**
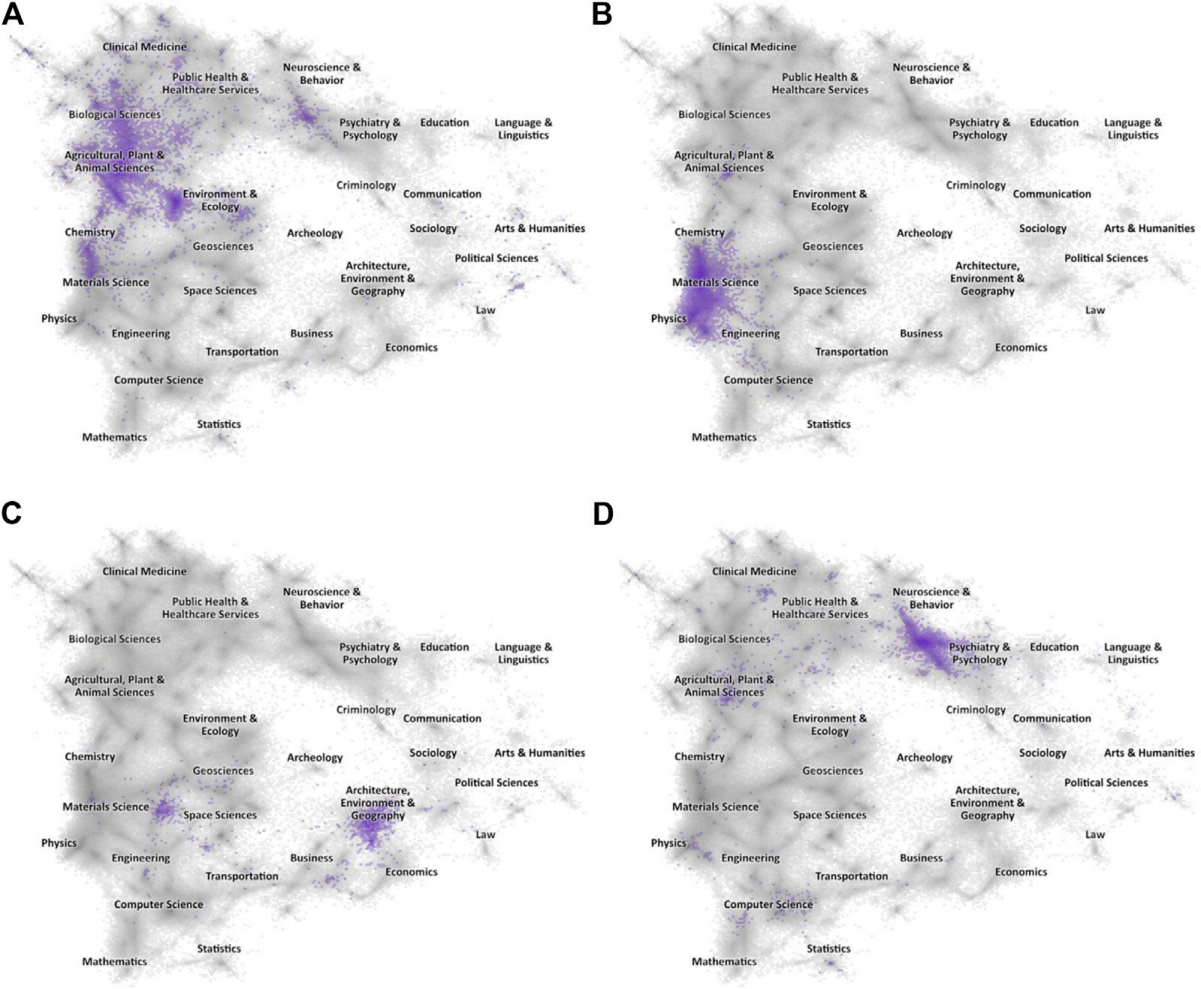
**(A)–(D)**. Science maps showing the spread of articles in four topical Research Fronts. The greyed landscape is the proximity map for all articles indexed in the Web of Science (2010–19) within which major domains have been labeled, and the highlighted area in each map are those papers linked to a specific topical Research Front. **(A)** CRISPR **(B)** 2D Materials. **(C)** Global Energy System Transition **(D)** Dynamic Functional Connectivity.

The approach to mapping scientific and scholarly research as traditionally employed at ISI and devised by Small is as follows: A research front appears when a set of recent publications all co-cite several earlier papers that stand out because they are themselves in the top 1% (the highest percentile class) for their year and field. The recent papers are linked by the highly-cited targets they cite in common and thus form an emerging front of research activity, the identification of which may be determined by a review of their common keywords (Figure 9).

For a research evaluator the first question is whether the work that they are reviewing appears in one or more of these research fronts. They can then use research fronts to address their knowledge of the additional issues of timeliness, which may be determined by the recency of the citing papers, and pervasiveness, which may be inferred by citation abundance and spread across fields.

More generally, for an institution, how much of its work is in or (extending the mapping analysis) close to a research front? Important management opportunities, which go far beyond the information derived from research performance metrics, appear when research fronts are precisely located in the knowledge network. A research manager can determine the distribution of institutional output across the knowledge landscape, filtering for recent or longer time windows, and then assess the relationship of their research clusters to a front. They can also make a comparative evaluation with competitor institutions. Similarly, research funders, by identifying the distribution of publications arising from funded projects, can see whether investments are producing work located in or near research fronts and policy makers can use this approach to map research emerging at a national level (Chinese Academy of Sciences, 2019; Igami and Saka, 2016).

### Context and Purpose

We started by noting that research evaluation is usually interested in excellence (Moore et al., 2017; Ferretti et al., 2018) and that “excellence depends on context” (Nature, 2018). The reach of and attention given to an innovation in an emerging cross-disciplinary research area will be very different to research with the direct but narrow impact critical to solving a technological constraint for an industrial process. What is true is that in both instances the research will only be ‘good, valid, timely and useful’ if it is high quality, yet that quality will not be measured by stakeholders in the same way.

Intention, purpose and objectives should be an embedded component of the initial design of every research assessment process. Why are we doing this, what do we seek to discover, what would tell us whether this research is good and what tells us whether it has achieved its aims? If an assessment starts without these criteria in mind and without adapting and matching the data, methods, analysis and indicators to those criteria then it is less likely to provide a satisfactory and informative interpretation of outcomes for the user.

We refer again to the perspectives listed by Langfeldt al (2020) and their relevance to the ABRC (1987) internal and external criteria. The values of novelty and utility are not the same thing. Both require ‘good’ research but the index of goodness for one may not be consonant with the other. Similarly, the value dimensions of researchers, research funders and national research policy will be conditioned by the objectives peculiar to each. The suitability of any bibliometric approach is proven by defining those objectives and setting the analysis in a structure that matches method to purpose.

### Discussion: Implications for the Original Example

There is a need for changes in the approach to using bibliometric data: the subject-expert user needs to be clear whether the data they have are relevant to the evaluation questions they pose; they need to establish an *a priori* understanding of how they will use the data and of the choices of methods to apply; and it should be standard practice that data are developed and presented not as summary point metrics but in a form that allows accessible, interpretive exploration through drilling down or ‘peeling the onion’ of any rich analysis.

It should be clear from this review of scientometric data underpinning bibliometric indicators that, when looking back at Table 1, an evaluator would be incautious if they were to rely solely on summary information to make judgments about the relative or absolute research strengths, even of whole countries. This should be even more true if they were reviewing a table of institutions from the same countries or a set of their research groups seeking funding, and yet this happens frequently.

Highly granular categorical systems group research papers into small, self-referential pockets that boost the apparent relative citation performance of work which appears poorly cited in familiar topical aggregations (Table 2). More generally, the effect of a choice of discipline/topic categories for aggregating publications and normalizing citations is two-fold. First, countries with a less developed domestic research base, and less well cited domestic research output, will tend to have smaller publication tallies when more exclusive categorical systems (such as ESI and the ANZSRC FORs) are used (Table 3). Second, because such categories focus on journals selectively, it is the least well cited part of a country’s activity that is omitted, so their average CNCI is raised (Figure 3). So, although publication counts for Sri Lanka, Bulgaria and Indonesia are significantly reduced in an ESI analysis compared with a WoS analysis they nonetheless then have higher average CNCI.

International collaboration is a pervasive factor for all countries and may cover much more than half their annual publication output, but the situation for smaller research economies is diverse (Figure 5). These collaborative papers are more highly cited on average, for all countries, and thus raise their average CNCI. For smaller countries, the balance of output and citations becomes disproportionate: for Indonesia 52% of papers are domestic but 88% citations come from international collaborative papers; for Sri Lanka the figures are 34% domestic papers and 90% international citations. Iran, by contrast, relies largely on its domestic research output. In consequence, Table 1 should be re-interpreted in the light of the balance of domestic and collaborative output and citations in each portfolio, and Figure 4 further emphasizes the potential benefit due to collaboration as compared to domestic activity.

Historical links to well established European research economies can have a significant research benefit because of sustained collaborative partnerships. This is an excellent outcome at a cultural and economic level but it could be a covert factor influencing outcomes at a bibliometric level. Argentina’s relationship with Spain and Indonesia’s relationship with the Netherlands are examples (Table 4).

Cultural factors are rarely identified as a research analytical factor at national level, although they are widely acknowledged at a gross (arts/science) and fine (molecular vs. organismal biology) disciplinary level. The beneficial effect of ‘recency’ on citation rates for Germany (and other G7 research economies) is apparent in comparison with China, which appears to cite later but then to have rising relative citation performance for any year (Figure 7). This highlights the need to be wary of any short windows in an analysis, or of focusing unduly on the most recent data, without understanding the research culture and behavior of the target under analysis.

A further complication with international collaboration and the relatively higher citation counts for international publications (Figure 4) is the consequent effect on net national CNCI. Every national portfolio is enhanced compared to the global pool because it contains only the national slice of lower cited domestic activity. The best way to interpret the real distribution of CNCI is through a graphical analysis that reveals the full profile, the balance of work above and below world average, and the components due to domestic and collaborative output. Ideally, this would include a relevant benchmark.

We wholly endorse the views of Moed (2020) regarding the need for an evaluation framework in which the context and the purpose of the exercise are over-riding considerations. Citations are themselves value-laden constructs with social as well as research weight. Any aggregation of citation counts, subsequent management of the data through normalization and fractionation, and choice of analytical methodology then applied, must introduce further subjective modification that moves from original information toward a stylized indicator. The reader is referred to Ferretti et al. (2018) for a discussion of the challenge in establishing consensus on indicators of excellence.

In summary, the points that we have reviewed and of which those users planning a research evaluation should be aware are:

Normalization, granularity: a choice of broad or narrow focus is made when citation counts are normalized against a global benchmark, for comparative purposes or to aggregate data across years and disciplines (Table 2).

• USERS need to be aware of granularity and choose an appropriate level of aggregation.

Normalization, categories: there are many systems for assigning journals and/or individual publications to discipline categories and none is uniquely correct (Table 3, Figure 3).

• USERS should take the assessee’s output portfolio into account in choosing a data source

Collaboration, domestic: the balance of domestic and internationally co-authored publications in a portfolio is likely to influence the evaluation outcome.

• USERS should be aware that papers with only domestic authors may be cited less often

Collaboration, impact: since internationally collaborative papers tend to higher citation impact the evaluator must reflect on the extent to which the data are driven by the target of evaluation or by work with its partners (Figure 6).

• USERS should consider the absolute and relative volume of international research collaboration

Collaboration, fractional attribution: it is argued that partitioning of credit for output and impact should be used to account for collaborative influence, but arithmetic solutions do not provably deliver greater precision or accuracy and are unlikely to assign the most appropriate fraction.

• USERS should be conscious of the balance of author counts in the evaluated output, and be aware of the effect of fractional attribution

History, legacy partners: the continuing influence of previous colonial relationships is evident (Table 4).

• USERS should recognize the legacy of history and consider how this might influence outcomes

Geography, distance and networks: not all countries are equal in their access to research partners by both distance and location.

• USERS should consider whether location factors may favor or constrain the assessed activity

Culture and language: there is a preference in some countries, sometimes stimulated by national Academies, to publish in nationally oriented journals and this, while entirely appropriate, naturally reduces exposure to external researchers who focus on ‘international’ journals.

• USERS should review the language balance in assessed output and any preference for journals with national rather than international orientation

Culture, national and disciplinary: differences in publishing and citing practice are known to exist between disciplines but it is less commonly acknowledged that distinctions in research culture also occur between countries (Figure 7).

• USERS should reflect on national and cultural components in data and indicators

Benchmark: the apparent anomaly that all nations can be above world average throws further light on the interpretation of trajectories (Figure 8).

• USERS must be sensitive to characteristics of the data and the analytical methods

Profiles: visualizing the full CNCI distribution in an Impact Profile not only shows the true spread of strong and weak performance around the average but also exposes the difference between that average and the median (Figure 4).

• USERS should seek data analyses that display the full distribution, not just point metrics

Context: most research indicators focus on a dataset for a target entity (country, institution, group) and the identification of research excellence. Research activity around the margins of that target and information in regard to other assessment criteria is less clear but it may be critical to interpretation and to the success of any intervention (Figure 9).

• USERS should consider that the research they assess is part of an ecosystem

The basic challenge for scientometrics is not about additional, new indicators but about presenting the outcomes of sound academic research in metrics and analytics in a form that domain specialist users can make use of for evaluation within their field. The future for the scientometrician should be less about the academic ideal in metrics, and its chimeric perfection, and more about user support including better management interpretation and faster, more confident decision making.

When the evaluator is clear about their objectives, the questions to be addressed, the relevance of bibliometrics to those questions and the nature of the available data, and the place of the bibliometric analysis within an overall evaluative framework, then they should proceed to work through the issues we list here and determine whether they have fully understood the implications of these and the outcome in the context of their purpose and materials. To facilitate such comprehension, this interpretation is preferably implemented locally, by the users (policy, funder, etc) and domain experts, rather than by an external analyst. The information presented must draw on a substantial body of data and may be best deployed not as tables but visualisations. It may also be that an intermediary - normally the secretariat supporting the decision-making group - is still required to mediate the interpretation. But this should now locate the target activity more closely for the evaluating group and in a meaningful context drawing on references to a wider information base that includes points familiar to multiple group members.

## Data Availability

The data analyzed in this study is subject to the following licenses/restrictions: Original data were sourced where indicated from the Web of Science, which is accessible to academic researchers in the United Kingdom under licence from the Joint Information Services Committee and in other countries through separate licensing agreements. Requests to access these datasets should be directed to https://clarivate.com/webofsciencegroup/solutions/web-of-science/contact-us/.
